# Pre-operative fear of falling is associated with worse 3-year mobility and quality of life in elderly hip fracture patients: a post-hoc analysis of a prospective cohort

**DOI:** 10.1186/s13018-026-06956-4

**Published:** 2026-06-01

**Authors:** Feng Gao, Shanbin Xu, Yimin Chen, Liunan Chen, Ruijiang Li, Yixiao Chen, Mingjian Bei, Tingrun Cui, Gang Liu, Minghui Yang, Xinbao Wu

**Affiliations:** 1 Department of Orthopaedics and Traumatology, Peking University Fourth School of Clinical Medicine, Beijing, China; 2 Department of Orthopaedics and Traumatology, Beijing Jishuitan Hospital, Capital Medical University, Beijing, China; 3 Department of Orthopaedics and Traumatology, National Center for Orthopaedics, Beijing, China

**Keywords:** Hip fracture, Fear of falling, Modified falls efficacy scale, Mobility

## Abstract

**Background:**

Hip fractures in older adults have become a global public health challenge. Such fractures not only carry a high mortality rate but also severely impair patients' mobility. Although it is known that fear of falling affects mobility recovery, existing evidence has largely focused on short-term outcomes within the first year post-surgery. The persistent impact of preoperative fear of falling on long-term mobility at three years post-surgery remains unclear. This study aims to evaluate the impact of preoperative fear of falling on mobility and health-related quality of life at three years postoperatively in elderly patients with hip fractures.

**Methods:**

A post-hoc analysis of a prospective study was done. Patients aged 65 years and above with hip fractures who underwent surgical treatment at Beijing Jishuitan Hospital between November 2018 and November 2019 were enrolled. Preoperative fear of falling was assessed using the Modified Fall Effectiveness Scale (MFES). Patients were categorized into low (MFES ≥ 8) and high (MFES < 8) fear groups. The primary outcome of this study was mobility at 3-year post-surgery. Secondary outcomes comprised mobility at 30-day, 120-day, and 1-year post-surgery, alongside quality of life at 30-day, 120-day, 1-year, and 3-year post-surgery.

**Results:**

A total of 683 patients were ultimately included in the analysis. There were statistically significant differences between the two groups in age, gender, educational level, hypertension, stroke, pre-fracture mobility, anesthesia method, and length of hospital stay (all *P* < 0.05). There were no significant differences in mobility or EQ-5D index between groups within first year post-surgery (all adjusted *P* > 0.05). However, statistically significant differences in mobility and EQ-5D index were observed between the two groups at the 3-year follow-up. Patients with low preoperative fear of falling demonstrated higher mobility and EQ-5D index at 3 years (all adjusted *P* < 0.001).

**Conclusion:**

The preoperative fear of falling was significantly associated with mobility and quality of life three years postoperatively in elderly hip fracture patients.

**Supplementary Information:**

The online version contains supplementary material available at 10.1186/s13018-026-06956-4.

## Background

Hip fractures in the elderly have become a global public health challenge. In 2021, the number of elderly individuals experiencing hip fractures due to falls increased by approximately 8.2 million compared to 30 years earlier [1]. Although the age-standardized incidence of hip fractures has shown a declining trend over time, the absolute number of cases continues to rise, largely driven by population aging and increased life expectancy. Consequently, the annual number of new hip fracture cases is projected to reach approximately 16.8 million worldwide by 2036, reflecting demographic expansion rather than an increase in individual risk [1]. Such fractures not only carry an extremely high mortality rate but also severely compromise patients' mobility [2–4]. Research indicates that only 40–60% of survivors regain pre-fracture mobility levels or functional independence, with approximately 18.7% remaining unable to walk independently one year post-surgery [5, 6]. Consequently, identifying predictable and modifiable indicators of functional outcomes is crucial for reducing long-term disability rates.

Among the numerous factors influencing functional recovery, fear of falling may play a critical role. In elderly hip fracture patients, the prevalence of fall fear can reach 47–68% [7, 8]. This fear is not merely a subjective concern and it is frequently accompanied by marked activity avoidance [9]. Reduced mobility leads to diminished muscle strength and endurance, subsequently compromising balance and gait stability [10]. Concurrently, prolonged inactivity may accelerate bone mass loss [11]. These cumulative changes readily form a vicious cycle of progressive deterioration. A meta-analysis by Ma et al. included seven cohort studies with 27,714 participants and demonstrated a significant positive association between fear of falling and worse outcome in middle-aged and older adults [12]. Previous studies have also indicated that fear of falling at 12 weeks post-surgery is significantly associated with poorer functional recovery at one year [7]. Furthermore, a comprehensive systematic review by Gadhvi et al. included 36 studies and 5099 participants, confirming that fear of falling after hip fracture is consistently associated with poorer physical function, including balance, gait speed, and self-reported function [13].

Although studies have reported an association between fear of falling and postoperative functional recovery, existing evidence predominantly focuses on short-term outcomes within 6 months to 1-year post-surgery [7, 14, 15]. Evidence remains lacking to determine whether fear of falling is sustainedly associated with functional outcomes three years post-surgery. Concurrently, research examining the relationship between preoperative fear of falling and functional recovery remains relatively scarce. If preoperative fear of falling could serve as an indicator of functional recovery risk, clinicians could identify high-risk individuals earlier during the perioperative period and implement targeted interventions promptly. This would help reduce activity avoidance and promote faster, more complete functional recovery. Therefore, this study aims to evaluate the predictive value of preoperative fear of falling on long-term postoperative mobility and quality of life.

## Materials and methods

### Study design and subjects

This study was a post-hoc analysis of a prospective study. Patients were drawn from elderly patients with hip fractures consecutively admitted to Beijing Jishuitan Hospital between November 2018 and November 2019. Inclusion criteria were: (1) Age ≥ 65 years; (2) Hip fracture confirmed by X-ray or CT (including femoral neck, intertrochanteric, or subtrochanteric fractures); (3) Time from injury to admission < 21 days; (4) Underwent surgical treatment. Exclusion criteria: (1) Pathological fractures; (2) Failure to sign informed consent.

The original study adheres to the Declaration of Helsinki and is registered on ClinicalTrials.gov (identifier: NCT03184896; registration date: June 14, 2017). Ethical approval was obtained from the institutional ethics committee. Informed consent was obtained from all individual participants included in the study.

### Exposure factor: preoperative fear of falling

Preoperative fear of falling was assessed using the modified Falls Efficacy Scale (MFES) to quantify patients' confidence levels in daily activities [16]. The MFES demonstrated excellent internal consistency in our study sample (Cronbach’s α = 0.980). Compared to the original 10-item FES, the MFES incorporates an assessment of outdoor activity capability, comprising 14 items related to daily living. Each item is scored using a 10-point visual analogue scale, and the overall MFES score is calculated as the average of the 14 item scores, ranging from 0 to 10, with higher scores indicating greater confidence. Patients were divided into two groups based on the cutoff score of 8, as suggested by previous research: MFES ≥ 8 constituted the low fear of falling group, and MFES < 8 constituted the high fear of falling group [17].

### Data collection and follow-up

Trained orthopedic nurses were responsible for patient screening, enrolment, and collection of baseline and follow-up data. Baseline information comprised: demographic details (age, gender, smoking history, alcohol history, residence, health insurance type, educational level, whether living alone); clinical and pre-injury status (fracture type, ASA grade, comorbidities, pre-fracture mobility); perioperative and hospitalization details (type of surgery, anesthesia method, length of hospital stay, total hospital costs). Follow-up assessments were conducted via telephone at 30-day, 120-day, 1-year, and 3-year post-admission to collect mobility and quality of life outcome measures. All interviewers (trained orthopedic nurses) received standardized training on the use of the questionnaire and on avoiding leading questions.

The primary outcome of this study was mobility at 3-year post-surgery. Secondary outcomes comprised mobility at 30-day, 120-day, and 1-year post-surgery, alongside quality of life at 30-day, 120-day, 1-year, and 3-year post-surgery. We categorized patients' postoperative mobility into three groups: independent walking, walking with a walking aid, and non-ambulant. Health-related quality of life was assessed using the EuroQol five-dimensional (EQ-5D) Questionnaire [18]. This scale describes health status across five dimensions: mobility, self-care, usual activities, pain/discomfort, and anxiety/depression. Each dimension comprises five possible states, ranging from “no problems” to “unable to do/extreme problems”. Health status across these five dimensions was converted into an EQ-5D index value using a standardized scoring algorithm [19].

### Statistical analysis

Continuous variables were presented as mean ± standard deviation or median (interquartile range) according to distribution characteristics. Categorical variables were expressed as counts (percentages). Comparisons between groups utilized t-tests or Mann–Whitney U tests for continuous variables and chi-square tests for categorical variables. Multivariate logistic regression models assessed associations between preoperative fear of falling and mobility at each follow-up time point. Using non-ambulant as the reference outcome, odds ratios (OR) and 95% confidence intervals were calculated for independent walking and walking with a walking. Multivariate linear regression models were employed to compare associations between preoperative fear of falling and EQ-5D scores at each follow-up time point, reporting regression coefficients and statistical test results. Multivariate analysis included clinically meaningful variable (MFES groups) and statistically significant variables (age, gender, educational level, hypertension, stroke, pre-fracture mobility, anesthesia method, and length of hospital stay). Inverse probability weighting (IPW) sensitivity analysis was conducted using baseline variables associated with follow-up completion (Appendix Table 1), and all outcome analyses were repeated with weighted regression. Statistical analysis was performed using the IBM SPSS Statistical Package (version 25) (SPSS Inc., Chicago, IL, USA). All statistical significance was established at *P* < 0.05.

**Table 1:** Comparison of baseline characteristics between two groups

	Total (n = 683)	Low fear group (n = 375)	high fear group (n = 308)	Statistics	*P* value
Age/ years, mean ± SD	79.01 ± 7.771	77.69 ± 7.438	80.62 ± 7.874	–5.003	< 0.001
Gender, n (%)				4.320	0.038
Female	492(72.0)	258(68.8)	234(76.0)	–	–
Male	191(28.0)	117(31.2)	74(24.0)	–	–
Smoking history, n (%)	107(15.7)	65(17.3)	42(13.6)	1.749	0.186
Alcohol drinking, n (%)	31(4.5)	19(5.1)	12(3.9)	0.535	0.465
Residence, n (%)				2.590	0.108
Rural area	52(7.6)	23(6.1)	29(9.4)	–	–
Urban area	631(92.4)	352(93.9)	279(90.6)	–	–
Insurance type, n (%)				1.449	0.717
Basic medical insurance	516(75.5)	280(74.7)	236(76.6)	–	–
New Rural Cooperative Medical Scheme	54(7.9)	28(7.5)	26(8.4)	–	–
Commercial insurance	4(0.6)	2(0.5)	2(0.6)	–	–
Other	109(16.0)	65(17.3)	44(14.3)	–	–
Educational level, n (%)				6.158	0.046
Primary school or below	277(40.6)	137(36.5)	140(45.5)	–	–
Middle/high school	311(45.5)	179(47.7)	132(42.9)	–	–
University or above	95(13.9)	59(15.7)	36(11.7)	–	–
Living alone, n (%)	71(10.4)	46(12.3)	25(8.1)	3.126	0.077
Fracture type, n (%)				2.222	0.317
Femoral neck fracture	356(52.1)	205(54.7)	151(49.0)	–	–
Intertrochanteric fracture	307(44.9)	160(42.7)	147(47.7)	–	–
Subtrochanteric fracture	20(2.9)	10(2.7)	10(3.2)	–	–
ASA grade, n (%)				3.698	0.054
Grade 1–2	452(66.2)	260(69.3)	192(62.3)	–	–
Grade 3–4	231(33.8)	115(30.7)	116(37.7)	–	–
Hypertension, n (%)	382(55.9)	195(52.0)	187(60.7)	5.210	0.022
Diabetes mellitus, n (%)	192(28.1)	105(28.0)	87(28.2)	0.005	0.943
Coronary heart disease, n (%)	136(19.9)	82(21.9)	54(17.5)	1.992	0.158
Stroke, n (%)	113(16.5)	49(13.1)	64(20.8)	7.285	0.007
Pre-fracture mobility, n (%)				50.101	< 0.001
Independent	505(73.9)	316(84.3)	189(61.4)	–	–
Walking aid	163(23.9)	50(13.3)	113(36.7)	–	–
Non-ambulant	15(2.2)	9(2.4)	6(1.9)	–	–
Type of surgery, n (%)				5.622	0.131
Cannulated screw fixation	77((11.3)	47(12.5)	30(9.7)	–	–
Intramedullary nailing	325(47.6)	171(45.6)	154(50.0)	–	–
Hemiarthroplasty	194(28.4)	101(26.9)	93(30.2)	–	–
Total hip arthroplasty	87(12.7)	56(14.9)	31(10.1)	–	–
Anesthesia method, n (%)				15.618	< 0.001
General anesthesia	28(4.1)	24(6.4)	4(1.3)	–	–
Neuraxial anesthesia	638(93.4)	338(90.1)	300(97.4)	–	–
Other	17(2.5)	13(3.5)	4(1.3)	–	–
Length of hospital stay, day, median (IQR)	4.96(3.96, 6.58)	4.88(3.92, 6.38)	5.13(4.08, 6.83)	-2.616	0.009
Total cost, thousand yuan, median (IQR)	54.07(46.13, 69.37)	53.65(45.94, 69.03)	55.92(46.80, 69.90)	-1.458	0.145

## Results

**Fig. 1:**
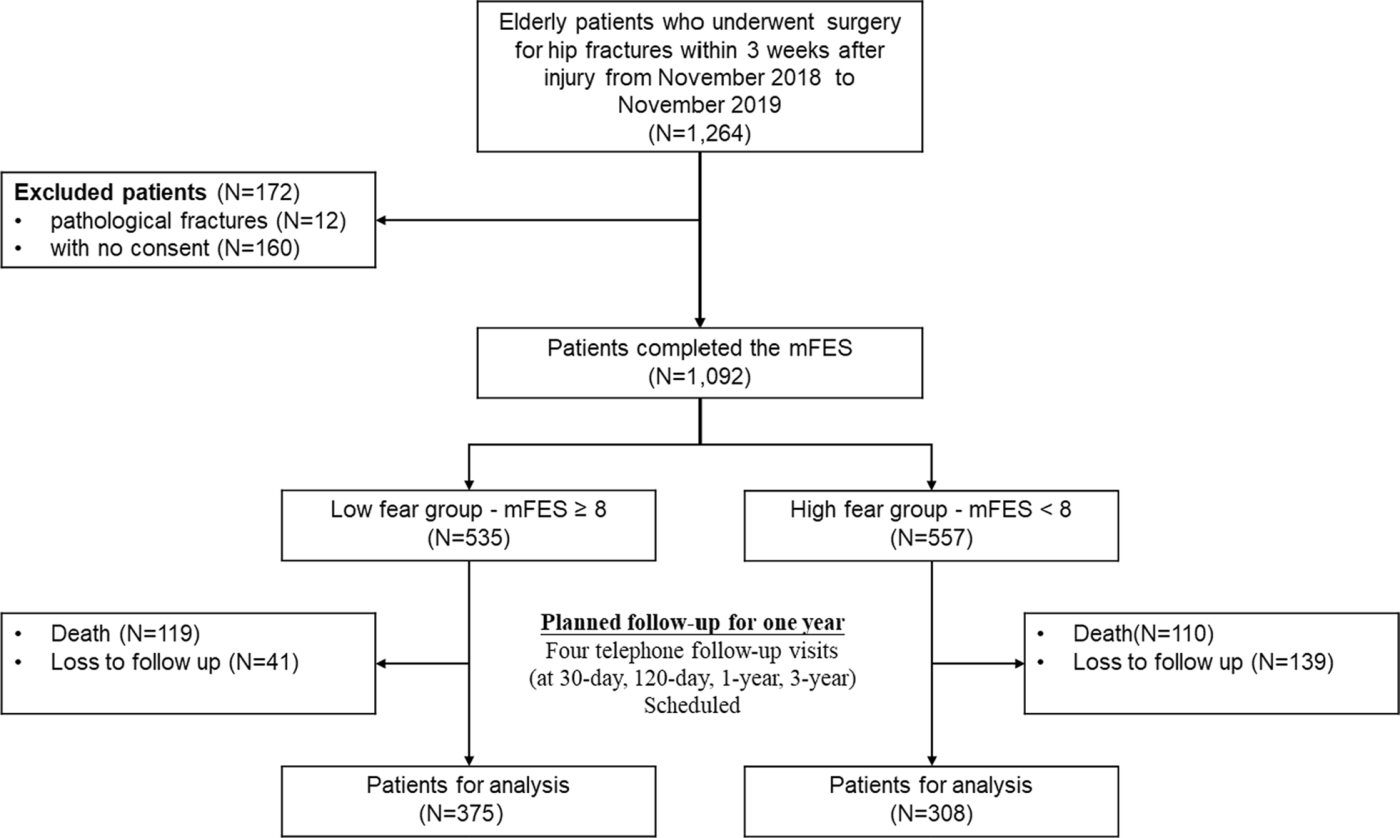
Flowchart

This study included 683 elderly hip fracture patients who completed the follow-up. Based on their MFES scores, 375 and 308 patients were assigned to the low (≥ 8) and high (< 8) fear groups, respectively (Fig. 1).

Patients in the high fear group were significantly older than those in the low fear group (80.62 ± 7.874 vs. 77.69 ± 7.438 years, *P* < 0.001). The proportion of female patients was also higher in the high fear group (76.0% vs. 68.8%, *P* = 0.038). Additionally, the high fear group showed significantly higher prevalence rates of hypertension (60.7% vs. 52.0%, *P* = 0.022) and stroke (20.8% vs. 13.1%, *P* = 0.007). Significant between-group differences were also observed in pre-fracture mobility, anesthesia method, and length of hospital stay (all *P* < 0.05). No statistically significant differences were found for the other baseline characteristics (all *P* > 0.05). The baseline characteristics of the two patient groups are presented in Table 1.

Table 2 presents postoperative mobility outcomes across follow-up periods. At 30-day after surgery, no significant differences in mobility were observed between the two groups in either univariate or multivariate analysis (all *P* > 0.05). At 120-day and 1-year postoperatively, univariate analysis indicated better mobility recovery in the low fear group compared to the high fear group (both *P* < 0.05). After multivariate adjustment for covariates, however, these differences were no longer statistically significant (all adjusted *P* > 0.05). By the 3-year follow-up, univariate analysis again showed superior mobility in the low fear group (*P* < 0.001). After adjustment, the low fear group remained significantly more likely to achieve independent walking (OR = 2.849, 95% CI: 1.553–5.228, adjusted *P* = 0.001) and assisted walking (OR = 2.486, 95% CI: 1.397–4.425, adjusted *P* = 0.002) compared to the high fear group.

**Table 2:** Comparison of postoperative mobility between two groups

	Total	Low fear group	High fear group	Statistics	*P* valu*e*	OR	95%CI	Adjusted *P* value*
30-day mobility, n (%)	–	–	–	5.549	0.063	–	–	–
Independent	55(8.1)	29(7.7)	26(8.4)	–	–	0.905	0.399 ~ 2.052	0.811
Walking aid	569(83.3)	322(85.9)	247(80.2)	–	–	1.313	0.714 ~ 2.414	0.382
Non-ambulant	59(8.6)	24(6.4)	35(11.4)	–	–	Ref	Ref	Ref
120-day mobility, n (%)	–	–	–	22.160	< 0.001	–	–	–
Independent	188(27.5)	129(34.4)	59(19.2)	–	–	1.737	0.692 ~ 4.361	0.240
Walking aid	465(68.1)	235(62.7)	230(74.7)	–	–	1.083	0.453 ~ 2.588	0.858
Non-ambulant	30(4.4)	19(6.2)	11(2.9)	–	–	Ref	Ref	Ref
1-year mobility, n (%)	–	–	–	8.341	0.016	–	–	–
Independent	368(53.9)	220(58.7)	148(48.1)	–	–	0.958	0.415 ~ 2.211	0.920
Walking aid	282(41.3)	141(37.6)	141(45.8)	–	–	1.160	0.508 ~ 2.647	0.725
Non-ambulant	33(4.8)	14(3.7)	19(6.2)	–	–	Ref	Ref	Ref
3-year mobility, n (%)	–	–	–	41.413	< 0.001	–	–	–
Independent	312(45.7)	206(54.9)	106(34.4)	–	–	2.849	1.553 ~ 5.228	0.001
Walking aid	290(42.5)	146(38.9)	144(46.8)	–	–	2.486	1.397 ~ 4.425	0.002
Non-ambulant	81(11.9)	23(6.1)	58(18.8)	–	–	Ref	Ref	Ref

*Multivariate analysis included MFES groups, age, gender, educational level, hypertension, stroke, pre-fracture mobility, anesthesia method, and length of hospital stay

Table 3 summarizes postoperative changes in EQ-5D index. In univariate analyses, the low fear group showed significantly higher EQ-5D scores than the high fear group at all time points (30-day, 120-day, 1-year, and 3-year; all *P* < 0.05). After multivariate adjustment, however, the differences at 30-day, 120-day, and 1-year were no longer statistically significant (all adjusted *P* > 0.05). In contrast, a significant between‑group difference remained at 3-year (adjusted* P* < 0.001). Higher preoperative fear of falling was significantly associated with a worse EQ‑5D index at 3-year after adjustment (b =  − 0.106, T =  − 4.105, adjusted *P* < 0.001).

**Table 3:** Comparison of postoperative EQ-5D index between two groups

	Total	Low fear group	High fear group	Statistics	*p* valu*e*	b	95%CI	Adjusted *P* value*
30-day, mean ± SD	0.64 ± 0.226	0.66 ± 0.199	0.62 ± 0.254	2.308	0.021	−0.011	−0.046 ~ 0.023	0.518
120-day, mean ± SD	0.79 ± 0.209	0.82 ± 0.194	0.75 ± 0.221	4.202	< 0.001	−0.110	−0.033 ~ 0.011	0.339
1-year, mean ± SD	0.84 ± 0.222	0.86 ± 0.208	0.81 ± 0.236	3.018	0.003	−0.019	−0.052 ~ 0.014	0.266
3-year, mean ± SD	0.66 ± 0.364	0.74 ± 0.312	0.56 ± 0.396	6.590	< 0.001	−0.106	−0.156 ~ -0.055	< 0.001

* Multivariate analysis included MFES groups, age, gender, educational level, hypertension, stroke, pre-fracture mobility, anesthesia method, and length of hospital stay

To examine whether attrition bias affected our findings, we repeated the analyses using inverse probability weighting (IPW) to adjust for differences between completers and non‑completers (Appendix Table 1). As shown in Table 4, the IPW‑weighted results were consistent with the primary completer‑only analysis.

**Table 4:** Inverse probability weighting (IPW) sensitivity analysis for mobility and EQ-5D index (30-day to 3-year)

	Original analysis (completers only) OR/b (95% CI) / *P*	IPW-weighted analysis OR/b (95% CI) / *P*
30-day mobility		
Independent	0.905 (0.399 ~ 2.052) / 0.811	0.918 (0.404 ~ 2.088) / 0.838
Walking aid	1.313 (0.714 ~ 2.414) / 0.382	1.296 (0.718 ~ 2.339) / 0.390
Non-ambulant	Ref	Ref
120-day mobility		
Independent	1.737 (0.692 ~ 4.361) / 0.240	2.058 (0.838 ~ 5.052) / 0.115
Walking aid	1.083 (0.453 ~ 2.588) / 0.858	1.246 (0.536 ~ 2.8944) / 0.609
Non-ambulant	Ref	Ref
1-year mobility		
Independent	0.958 (0.415 ~ 2.211) / 0.920	1.122 (0.497 ~ 2.530) / 0.782
Walking aid	1.160 (0.508 ~ 2.647) / 0.725	1.335 (0.599 ~ 2.974) / 0.480
Non-ambulant	Ref	Ref
3-year mobility		
Independent	2.849 (1.553 ~ 5.228) / 0.001	3.149 (1.730 ~ 5.732) / < 0.001
Walking aid	2.486 (1.397 ~ 4.425) / 0.002	2.756 (1.570 ~ 4.837) / < 0.001
Non-ambulant	Ref	Ref
30-day EQ-5D index	−0.011 (−0.046 ~ 0.023) /0.518	−0.012(−0.047 ~ 0.023) /0.498
120-day EQ-5D index	−0.110 (−0.033 ~ 0.011) /0.339	−0.011(−0.033 ~ 0.012) /0.362
1-year EQ-5D index	−0.019 (−0.052 ~ 0.014) /0.266	−0.021(−0.055 ~ 0.014) /0.237
3-year EQ-5D index	−0.106 (−0.156 ~ -0.055) / < 0.001	−0.116(−0.168 ~ -0.064) / < 0.001

## Discussion

This study systematically assessed the impact of preoperative fear of falling on the prognosis of elderly patients with hip fractures. Analysis of baseline data revealed that high preoperative fear of falling was significantly associated with advanced age, female gender, lower educational level, hypertension, stroke, reduced pre-fracture mobility, more neuraxial anesthesia, and longer length of hospital stay. Key findings indicated that the preoperative fear of falling was an independent risk factor of mobility and health-related quality of life at 3-year postoperatively. After adjusting for all covariates, the low fear group demonstrated a 2.8-fold greater likelihood of achieving independent walking and a 2.5-fold greater likelihood of achieving assisted walking at 3-year post-surgery compared to the high fear group. Furthermore, lower preoperative fear of falling corresponded with significantly improved EQ-5D scores at 3-year post-surgery. Nevertheless, the observed odds ratios are moderate, and residual confounding from unmeasured factors cannot be fully excluded; therefore, these findings should be interpreted with caution.

This study's multivariate analysis revealed that preoperative fear of falling had no independent association on mobility within the first year post-surgery. This finding contrasts with the results of studies by Oude Voshaar and Bower et al. [7, 15]. Oude Voshaar found that high levels of fear at 6 weeks postoperatively significantly reduced functional levels at 6 months. Bower, meanwhile, confirmed that high fear at twelve weeks postoperatively significantly diminished recovery levels at one year.

This discrepancy stems from the differing nature of the assessment time points. The 6–12-week postoperative period represents a critical phase when patients attempt community reintegration [20]. Persistent significant fear following over two months of professional rehabilitation typically indicates a stagnation in the recovery process. At this stage, the fear is no longer merely a psychological issue but serves as feedback of functional impairment. Consequently, the psychological state at this time point directly reflects short-term functional limitations.

By contrast, the preoperative assessment employed in this study reflects patients' psychological baseline. When patients entered the hospital, all patients in this study entered a geriatric hip fracture co-management model. Under this framework, intensive rehabilitation training by therapists, coupled with family support, provided robust external assistance [21, 22]. This external aid compensated for the lack of proactive motivation among high fear patients in the short term [23]. Consequently, the negative impact of preoperative psychological efficacy was masked by intervention measures and physiological recovery, failing to translate into significant mobility decline.

Over time, initial medical support and intensive home care gradually diminish. Patients enter a phase where maintaining activity relies entirely on personal initiative. At this stage, the intense pre-existing fear of falling is associated with cumulative functional decline. Patients with high fear levels persistently restrict independent activity due to instinctive apprehension [24]. This prolonged behavioral inhibition ultimately is associated with loss of muscle strength and functional decline [25]. Consequently, by the third-year follow-up, patients' functional status exhibits marked decline. This hypothesized pathway—from activity restriction to muscle atrophy and functional decline—is speculative and requires confirmation in future studies that incorporate serial objective physical function assessments.

This study confirms that preoperative fear of falling is independently associated with quality of life at 3-year post-surgery. Higher MFES scores correlate with better EQ-5D index at the 3-year post-surgery. The observed association (b =  − 0.106) exceeds the reported minimal clinically important difference (MCID) for the EQ-5D index, which is typically in the range of 0.03–0.07 [26]. This suggests that the impact of preoperative fear of falling on long-term health-related quality of life is not only statistically significant but also clinically meaningful. However, this differs from the findings of Beeres et al. [27]. It should be noted that Beeres' assessment occurred 1-year post-surgery, revealing a strong correlation between contemporary fear levels and quality of life at that time. In contrast, our study focuses on the impact of preoperative baseline factors. During the first year of this study, no significant differences were observed, which further suggests the protective role of the collaborative management model and family care. Strong external support temporarily compensated for the psychological deficiencies and the negative impacts brought by fear. However, at the three-year follow-up stage, this effect weakened. This was associated with dual functional and psychological impairment, with differences ultimately becoming apparent. Focusing solely on short-term recovery may overlook the risk of long-term decline in quality of life. In the overall cohort, the EQ-5D index declined from 0.84 at 1 year to 0.66 at 3 years, which is likely attributable to age‑related comorbidity accumulation and reduced social support over time. Importantly, despite this general decline, the low fear group maintained significantly higher EQ-5D scores at 3 years, indicating that preoperative fear of falling is associated with long‑term quality of life independent of these factors.

This study possesses the following strengths: Firstly, the follow-up period extended to three years. Current research on fear of falling predominantly focuses on the first year post-surgery, and this study effectively fills the evidence gap regarding its long-term mobility. Secondly, the assessment timing was early. This study emphasizes the influence of preoperative psychological baseline, aiding clinicians in identifying high-risk individuals during the early perioperative period. Finally, the substantial sample size. The study ultimately enrolled 683 elderly patients, enhancing statistical power and the robustness of the findings. At the same time, this study also had certain limitations. Firstly, this study design is prospective but not randomized, and it was conducted at a single tertiary hospital in Beijing with a predominantly urban sample (92.4%), which may restrict the generalizability of findings across different regions. Secondly, substantial patient attrition due to death or loss to follow-up during the three-year observation period may introduce selection bias. Thirdly, although multivariate regression analysis accounted for key variables such as age and underlying medical conditions, unmeasured psychosocial factors (including cognitive function, depression, and anxiety) may still confound the final outcomes. Fourthly, the MFES cutoff of 8 has not been formally validated in Chinese hip fracture patients, which should be considered when interpreting our findings. Fifthly, although we used a standardized questionnaire and trained interviewers to avoid leading questions, telephone follow‑up may still introduce recall bias and social desirability bias, potentially affecting the accuracy of self‑reported mobility and EQ-5D assessments. In addition, formal inter‑rater reliability testing for the telephone follow‑up assessments was not performed, although all interviewers received uniform training. Sixth, pre‑fracture mobility was assessed by patient or proxy recall, which may introduce some recall bias despite the short recall period (≤ 21 days).

## Conclusion

The preoperative fear of falling was significantly associated with mobility and quality of life three years postoperatively in elderly hip fracture patients.

## Supplementary Information

Below is the link to the electronic supplementary material.

Supplementary file1 (DOCX 23 kb)

## Data Availability

The dataset is managed by Beijing Jishuitan Hospital. The data access request can contact the corresponding author.

## Abbreviations

MFES: Modified fall effectiveness scale
ASA: American Society of Anesthesiologists
EQ-5D: EuroQol five-dimensional
OR: Odds ratio
95% CI: 95% Confidence interval
SD: Standard deviation
IQR: Interquartile range
IPW: Inverse probability weighting
