# Supplementary material for: Pre-operative fear of falling is associated with worse 3-year mobility and quality of life in elderly hip fracture patients: a post-hoc analysis of a prospective cohort
Source: J Orthop Surg Res. 2026 Jun 1;21:471. doi: 10.1186/s13018-026-06956-4 (PMC13439894; doi:10.1186/s13018-026-06956-4)
Supplement: Supplementary file 1 [file 13018_2026_6956_MOESM1_ESM.docx]

Appendix Table 1. Baseline characteristics of follow-up completers and non-completers

|  | Completers (n=683) | Non-completers  (n=409) | *P* value |
| --- | --- | --- | --- |
| Age/ years, mean±SD | 79.01±7.771 | 82.12±7.438 | <0.001 |
| Gender, n (%) |  |  | 0.957 |
| Female | 492(72.0) | 293(71.9) |  |
| Male | 191(28.0) | 115(28.1) |  |
| Smoking history, n (%) | 107(15.7) | 76(18.6) | 0.212 |
| Alcohol drinking, n (%) | 31(4.5) | 19(4.6) | 0.935 |
| Residence, n (%) |  |  | 0.266 |
| Rural area | 52(7.6) | 39(9.5) |  |
| Urban area | 631(92.4) | 370(90.5) |  |
| Insurance type, n (%) |  |  | 0.137 |
| Basic medical insurance | 516(75.5) | 302(73.8) |  |
| New Rural Cooperative Medical Scheme | 54(7.9) | 34(8.3) |  |
| Commercial insurance | 4(0.6) | 9(2.2) |  |
| Other | 109(16.0) | 64(15.6) |  |
| Educational level, n (%) |  |  | 0.025 |
| Primary school or below | 277(40.6) | 198(48.4) |  |
| Middle/high school | 311(45.5) | 154(37.7) |  |
| University or above | 95(13.9) | 57(13.9) |  |
| Living alone, n (%) | 71(10.4) | 58(14.2) | 0.061 |
| Fracture type, n (%) |  |  | 0.081 |
| Femoral neck fracture | 356(52.1) | 200(48.9) |  |
| Intertrochanteric fracture | 307(44.9) | 204(49.9) |  |
| Subtrochanteric fracture | 20(2.9) | 5(1.2) |  |
| ASA grade, n (%) |  |  | <0.001 |
| Grade 1-2 | 452(66.2) | 223(54.5) |  |
| Grade 3-4 | 231(33.8) | 186(45.5) |  |
| Hypertension, n (%) | 382(55.9) | 241(58.9) | 0.333 |
| Diabetes mellitus, n (%) | 192(28.1) | 111(27.1) | 0.728 |
| Coronary heart disease, n (%) | 136(19.9) | 108(26.4) | 0.013 |
| Stroke, n (%) | 113(16.5) | 81(19.8) | 0.173 |
| Pre-fracture mobility, n (%) |  |  | <0.001 |
| Independent | 505(73.9) | 261(63.8) |  |
| Walking aid | 163(23.9) | 143(35.0) |  |
| Non-ambulant | 15(2.2) | 5(1.2) |  |
| Type of surgery, n (%) |  |  | 0.049 |
| Cannulated screw fixation | 77((11.3) | 36(8.8) |  |
| Intramedullary nailing | 325(47.6) | 207(50.6) |  |
| Hemiarthroplasty | 194(28.4) | 132(32.3) |  |
| Total hip arthroplasty | 87(12.7) | 34(8.3) |  |
| Anesthesia method, n (%) |  |  | 0.565 |
| General anesthesia | 28(4.1) | 12(2.9) |  |
| Neuraxial anesthesia | 638(93.4) | 385(94.1) |  |
| Other | 17(2.5) | 12(2.9) |  |
| Length of hospital stay, day, median (IQR) | 4.96(3.96, 6.58) | 5.13(4.08, 6.60) | 0.165 |
| Total cost, thousand yuan, median (IQR) | 54.07(46.13, 69.37) | 56.18(49.17, 67.14) | 0.122 |
